# Response to “Randomized Controlled Trials on COVID-19 Should be Accurate and Trustworthy”

**DOI:** 10.4269/ajtmh.21-0836b

**Published:** 2021-08-30

**Authors:** Sherief Abd-Elsalam, Shaimaa Soliman

**Affiliations:** Tropical Medicine and Infectious Diseases Department Faculty of Medicine, Tanta University Tanta, Egypt E-mail: sherif.abdelbaky@med.tanta.edu.eg; Public Health and Community Medicine Menoufia University Menoufia, Egypt E-mail: shaimaasherif@hotmail.com

Dear Sir,

We thank Ben W. Mol for his comments and questions,^1^ and appreciate the meticulous reading of our recently published article.^2^ We agree that randomized controlled trials on COVID-19 should be accurate and trustworthy.

Regarding the first concern, our study started on March 23 and was finalized on June 1, 2020. Patients were monitored for 4 weeks. All data were available by July 1, and we worked to publish our report as quickly as possible to help address controversies regarding use of hydroxychloroquine for COVID-19. We are proud that our study was among the early studies that found that hydroxychloroquine has no benefit in the management of COVID-19.^2^^,^^3^

Regarding the second concern about conducting two trials during the same period,^4^^,^^5^ we would like to clarify that we contributed to the multicenter study published in the *Archives of Virology*, but not the other referenced study. Regarding the third concern, updated information is provided in the clinical trial registry. Also, the sample size determination was provided in the Methods section, and a post hoc sample power analysis was included at the end of the Results section.^2^

Regarding the fourth concern, most variables were nonparametric, and *P* values were calculated with the Mann Whitney test; it is not clear how *P* values could be calculated without the data rank. Regarding the fifth concern, we included median values for some of the nonparametric variables, and this might have caused confusion. Regarding the last concern, the recovery rate is a categorical variable analyzed by the χ^2^ or Fisher exact test, whereas the other variables considered were continuous and analyzed using the *t*- or Mann Whitney tests. In our case, the *P* value for comparing disease severity between groups was 0.06, and all other *P* values were insignificant as well.

We again thank Ben W. Mol for his great interest in our article.
